# Analysis of Compliance with Time under Tension and Force during Strengthening Exercises with Elastic Bands

**DOI:** 10.3390/diagnostics11112016

**Published:** 2021-10-29

**Authors:** Javier González-Rosalén, Francesc Medina-Mirapeix, Alba Cuerda-Del Pino, Noemi Moreno-Segura, Mariano Gacto-Sánchez, Rodrigo Martín-San Agustín

**Affiliations:** 1 Department of Physiotherapy, University of Valencia, CP 46010 Valencia, Spain; javigonros96@gmail.com (J.G.-R.); cuerda@alumni.uv.es (A.C.-D.P.); noemimorenosegura@gmail.com (N.M.-S.); 2 Department of Physiotherapy, University of Murcia, CP 30100 Murcia, Spain; mirapeix@um.es (F.M.-M.); marianogacto@um.es (M.G.-S.)

**Keywords:** elastic band, adherence, time under tension, strength training

## Abstract

Quantifying training variables of a physical exercise modality is essential for an appropriate dosage. In training with elastic bands, time under tension (TUT) and force represent the duration and intensity of this force-training modality. The aims of this study were to evaluate the degree of compliance to TUT prescription for three different scenarios of two exercises and the comparison of the force values obtained versus the estimate values. A total of 29 healthy volunteers were evaluated in a clinical environment under controlled conditions in 3 different scenarios (different velocities or ROMs) of both shoulder abduction and knee extension in 2 sets of 10 repetitions per scenario within a single session. Concentric and isometric phases showed a higher degree of compliance for their TUTs than the eccentric phase TUTs for all scenarios of both exercises, whereas the degree of compliance was higher for the total TUT than for the phases’ TUTs. Additionally, the eccentric phase showed a general tendency to develop for longer time periods than prescribed, whilst the fast scenario showed a higher degree of compliance for isometric phase TUTs and total TUTs than the extant two scenarios in both exercises. On the other hand, the force of the elastic bands tends to be overestimated according to the estimates of the manufacturers. These findings, both those related to the degree of compliance with TUTs and the force analysis, can be used by physiotherapists and other exercise professionals as a reference to achieve a good dosage of routine exercises with elastic bands.

## 1. Introduction

Physical exercise has been widely implemented in different fields, ranging from training to rehabilitation of injuries [1,2]. In multiple pathologies, physical interventions are a primary strategy included in clinical guidelines for the management of the disease, whether in acute, subacute, or chronic musculoskeletal injuries [3,4,5]. In addition, exercise has been proposed as an adjunctive treatment recommendation in other conditions in which it exerts the function of second-line treatment and improves the course of the pathology [6] as in type 1 diabetes [7,8,9], cancer survivors [10], fibromyalgia [11,12,13], or patients undergoing long-term haemodialysis [14,15,16].

Aerobic endurance exercise and resistance training have been traditionally implemented as training modalities in rehabilitation programs [1,2,17]. Although both can promote substantial benefits when appropriately prescribed, most of the evidence supports the inclusion of resistance training ahead of endurance training in current recommendations and guidelines [6,17,18]. Even though several studies confirm the health benefits of resistance training, physical activity on individuals with pathology should be individually tailored to prevent adverse reactions [1]. Thus, aspects of physical exercise in relation to prescription such as the type, frequency, intensity, and duration are critical in the implementation of exercise in a clinical environment [1,6]. The management of the aforementioned training variables in a clinical setting is usually developed by physiotherapists for a specific purpose, based on each patient’s condition [6,18].

Exercise interventions are usually divided into supervised gym-based programs and home-based training programmes [19]. Gym-based programmes may have an advantage over home-based programmes by controlling the amount and quality of direct training and supervision. However, following a home-based rehabilitation programme promotes the acquisition of a more active role in patients and improves the attachment to physical activity, along with behavioural change techniques [6,18,19].

Among the different methods of applying load in resistance training in home-based programmes, elastic bands have proven to be effective in the rehabilitation of shoulder, neck, knee, and hip pain [20]. Home-based rehabilitation is one of the potential beneficiaries of the implementation of elastic bands in training due to their low price, adaptability to different environments, and simple ability to progress [9,21]. Together with type, frequency, and intensity, time under tension (TUT) is a specific and important training variable in elastic band exercises. Total TUT reflects the time component of a strengthening exercise and refers to the sum of concentric, quasi-isometric, and eccentric contraction phases in a single training set [20,22,23]. Previous research on elastic band rehabilitation programs has proposed three different scenarios related to commonly prescribed home strengthening exercises, which represent either explosive, traditional strength exercises, or strength exercises where the full range of motion (ROM) cannot be obtained [20,23,24]. Despite the fact that the benefits of a training programme depend directly on the degree of compliance with the prescription, the study of prescribed TUTs in different exercises and scenarios has not been previously evaluated. Moreover, knowing the tension (i.e., intensity) that the elastic band performs is also essential for an appropriate dosage. Thus, although manufacturers usually provide tension values according to the percentage of elongation of the elastic band, several authors have shown that these are not usually accurate after a laboratory analysis [25]. Therefore, the first objective of this study was to analyse TUTs during different exercises and scenarios to evaluate the degree of compliance of the subjects for the prescribed scenarios. A second objective was to evaluate the tension obtained during these scenarios and to compare it with the estimated values.

## 2. Materials and Methods

### 2.1. Experimental Approach to the Problem

A cross-sectional study design was used to determine TUT and force parameters of elastic band training for shoulder abduction and knee extension. Each exercise was performed in three different scenarios (different velocities or ROMs), in 2 sets of 10 repetitions per scenario. All measurements were made in a single session under similar conditions of temperature (21 °C) and light in the clinical research laboratory of the Department of Physiotherapy (University of Valencia, Valencia, Spain).

### 2.2. Subjects

A total of 29 healthy volunteers (24 males; mean age: 23.6 ± 2.9 years; body mass: 75.8 ± 11.3 kg; stature: 176.4 ± 6.6 cm; weekly physical activity: 413.4 ± 179.5 min) who were recreationally active (engaging in 1–5 h of moderate physical activity 3–4 days per week) [26] were evaluated; all of them were students from the University of Valencia. All participants practiced recreational sports such as running, swimming, cycling, or general strength training. Subjects with injuries, diseases, or pain preventing proper exercise were excluded. The experimental protocol was approved by the Ethics Committee of the University of Valencia (Spain) (1239215). Once the study procedures were explained to the participants in detail, they signed informed consent and completed the demographic information sheet prior to data collection. 

### 2.3. Procedures

Before measurements, subjects were requested not to participate in any strenuous exercise during the previous 48 h. The evaluation session started with a standard warm-up, which consisted of walking up and down several flights of stairs and performing weight-free shoulder abductions. Shoulder abduction and knee extension with elastic bands were evaluated in three common elastic band training scenarios: (I) 0–90° at high velocity, (II) 0–90° at low velocity, and (III) 0–45° at low velocity [20]. These scenarios have been proposed to represent either (I) explosive, (II) traditional strength exercises, or (III) strength exercises where full ROM cannot be obtained, as it is often stated in patients with severe shoulder impingement [20]. In turn, each scenario has a set time associated with each one of the 3 movement phases (concentric, isometric, eccentric) and a rest time between repetitions as follows: (I) 1/2/1/1 s, (II) 3/2/3/2 s, and (III) 1.5/2/1.5/2 s [20]. The velocity of execution of each scenario was provided to the participants through feedback from a metronome. Furthermore, the participants were verbally guided if they did not follow the beat of the metronome.

**Figure 1:**
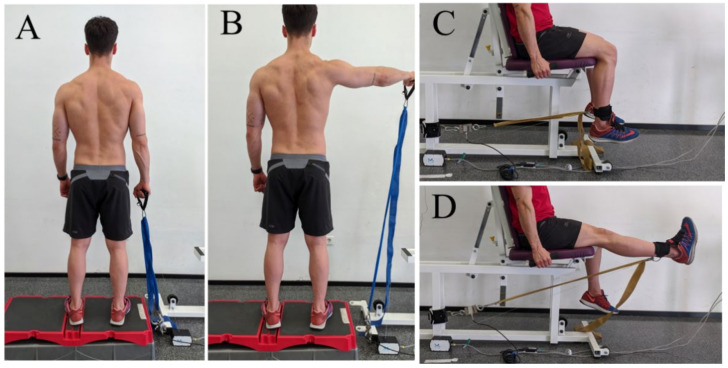
Shoulder abduction setup (**A**,**B**) using a force gauge anchored to the traction end of the elastic band and a linear encoder to assess velocity and elongation. Starting point in a step to avoid the height of the force gauge. Knee extension setup (**C**,**D**) using the same procedure, starting at 90° of hip and knee flexion, reaching maximum knee extension, and ending in the starting point.

The guidelines established for performing shoulder abduction were proposed in previous studies [27] as follows: (I) hip-width distance between the feet, (II) 30° horizontal flexion, (III) palm facing the floor, and (IV) slight elbow flexion. For knee extension, subjects sat on a quadriceps extension machine with a 90° hip flexion and an elastic band perpendicularly anchored to the ankle five cm above the malleoli (Figure 1). The order of the exercises and scenarios was randomised using a randomised number system for both exercises and scenarios. 

The elastic bands used were TheraBand CLX (The Hygenic Corporation, Akron, OH, USA): blue for abduction and golden for extension with a length of 40 cm (i.e., 2 loops), which are commonly used in rehabilitation studies [28,29,30]. The traction end of the elastic band was anchored to either a handle for abduction or an ankle brace for extension. The fixed end of the elastic band was anchored to a force gauge (MuscleLab 4020e, Ergotest Technology AS, Porsgrunn, Norway) (Figure 1). Since the shoulder abduction exercise is generally performed by stepping on the elastic band [20,24,27], the subjects were placed in a step to avoid the height of the force gauge. The movement velocity and elongation length of the elastic band were evaluated using a linear encoder (MuscleLab 4020e, Ergotest Technology AS, Porsgrunn, Norway) anchored to the handle or ankle (Figure 1). The participants had two familiarisation attempts per scenario. With a slight tension of the elastic band in the starting position (i.e., the minimum to avoid wrinkles in the elastic band), the subjects had to perform 2 sets of 10 repetitions per exercise and scenario, with 2 min of rest between sets. 

**Figure 2:**
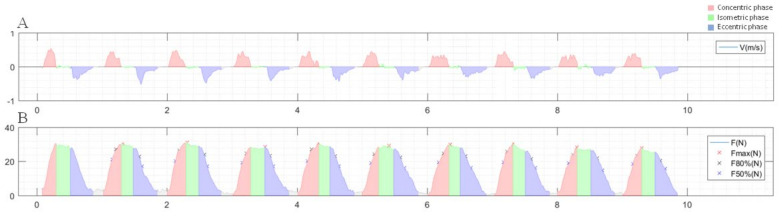
Graph of the information processed by MATLAB for a series of 90° shoulder slow abduction: (**A**) identification of the phases according to the velocity: red (positive) for the concentric phase, green (close to 0 m/s) for the isometric phase, and blue (negative) for the eccentric phase; (**B**) parameters of force by repetition and movement phases (example of slow shoulder abduction).

The linear encoder and force gauge information was recorded by the Data Synchronisation Unit (DSU) ML6000 (MuscleLab 4020e, Ergotest Technology AS, Porsgrunn, Norway) [31], which is the unit where the MuscleLab 4020e sensors are connected and integrated. Movement phases and TUTs and force parameters were calculated using custom-written scripts computed with MATLAB (version R2019b; The Mathworks, Natick, MA, USA). The phase of the movement for each repetition was determined from the velocity: positive for the concentric phase, around 0 m/s for the isometric phase, and negative velocity for the eccentric phase (Figure 2).

Once the phases were determined, the TUT and force parameters were calculated. The TUTs calculated were concentric TUT, isometric TUT, eccentric TUT, and single repetition TUT as the sum of concentric, isometric, and eccentric TUT during 1 repetition. The analysed force parameters (Newton) were tension at 50% and 80% in each phase (measured as 50% and 80% of the maximum elongation of each phase, since the elongation at the end of the concentric phase was generally greater than the beginning of the eccentric phase) and maximum tension obtained (Figure 2A). The means across the 10 repetitions for each parameter were calculated. Furthermore, based on the elastic band elongation percentage, the maximum estimated force for each exercise and scenario was calculated, based on the information supplied by the manufacturer [32]. Elastic band elongation was calculated by subtracting the initial length at rest (i.e., 40 cm) from the length of the elastic band at the end of the ROM (measured with the linear encoder). 

### 2.4. Statistical Analysis

Participant characteristics, force, and TUTs (Newtons (N) or seconds (s)), respectively, for each phase are presented as average (SD) and 95% confidence intervals (CIs). Mean between series was used for analysis. The degree of compliance with the expected TUTs per phase and total was analysed by subject and repetition, considering the obtained TUT as ‘fulfilled’ whenever the error was below 10% with respect to the prescribed TUT. This analysis is shown by frequencies; TUTs of the repetitions were subsequently either in range, exceeded the prescribed TUT, or did not reach the prescribed TUT.

Paired *t*-tests were used to analyse differences between the maximum force obtained and the maximum estimated force per scenario and exercise, and Cohen’s *d* was calculated to evaluate the effect size (d > 0.2: trivial, 0.2–0.5: small, 0.5–0.8: medium, and >0.8: large) [33]. All analyses were performed using SPSS (version 25; SPSS Inc, Chicago, IL, USA).

## 3. Results

### 3.1. TUTs

Table 1 shows average TUT values for the fast, slow, and restricted ROM scenarios for shoulder abduction and knee extension. The mean of total TUTs per repetition in the fast abduction scenario was 4.07 s. Contraction phase means were 0.95 s for concentric, 1.92 s for isometric, and 1.19 s for eccentric TUTs representing, respectively, 24%, 47%, and 29% of the total TUT. The slow abduction scenario showed a mean of total TUT of 8.5 s. Contraction phase percentages were 34% for concentric, 21% for isometric, and 45% for eccentric of the total TUT. The mean corresponding to total TUTs of restricted abduction ROM scenario was 5.45 s. Contraction phase percentages were 30% for concentric, 33% for isometric, and 37% for eccentric of the total TUT. The mean TUT values obtained on the contraction phase for knee extension scenarios were very similar to those obtained in shoulder abduction scenarios. 

#### 3.1.1. Shoulder Abduction

**Figure 3:**
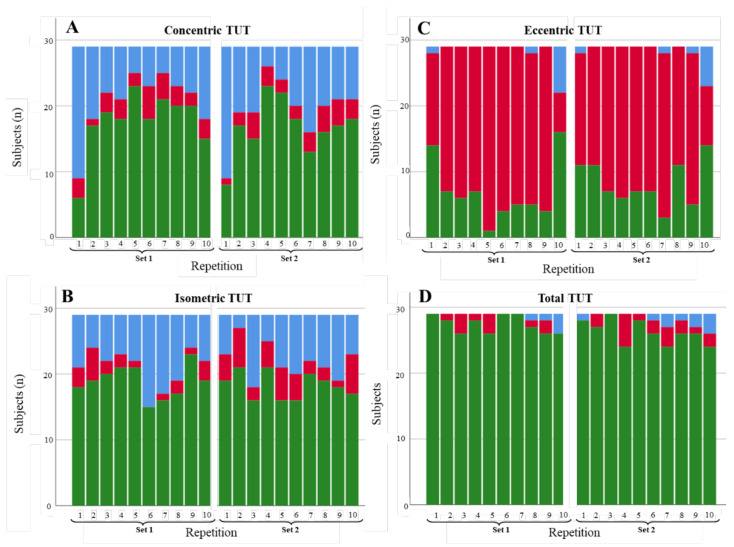
Degree of compliance of the TUTs for the fast abduction scenario by contraction phase: (**A**) concentric, (**B**) isometric, (**C**) eccentric, and (**D**) total. TUTs: in range = green; performed in less time than expected = blue; performed in more time than expected = red.

**Figure 4:**
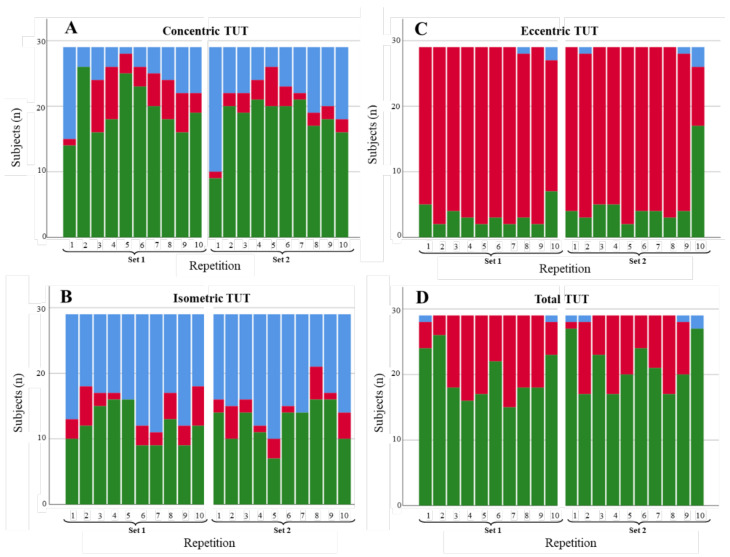
Degree of compliance of the TUTs for the slow abduction scenario by contraction phase: (**A**) concentric, (**B**) isometric, (**C**) eccentric, and (**D**) total. TUTs: in range = green; performed in less time than expected = blue; performed in more time than expected = red.

**Figure 5:**
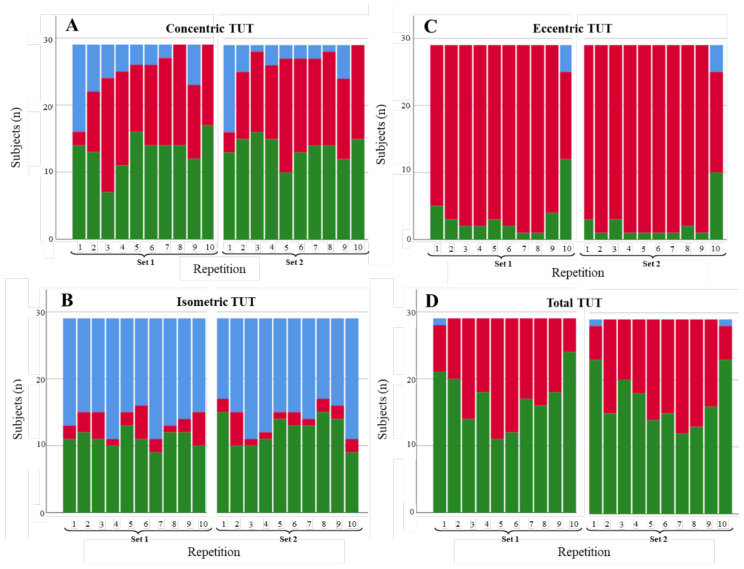
Degree of compliance of the TUTs for the restricted abduction ROM scenario by contraction phase: (**A**) concentric, (**B**) isometric, (**C**) eccentric, and (**D**) total. TUTs: in range = green; performed in less time than expected = blue; performed in more time than expected = red.

Figure 3, Figure 4 and Figure 5 illustrate the degree of compliance of TUTs in each repetition per contraction phase for the three shoulder abduction scenarios. In the concentric phase of the fast abduction scenario (Figure 3A), 344 of 580 of the repetitions were in range (59.3%), although the degree of compliance was lower in the first repetition of each set (6 and 8 of 29 repetitions in range in set 1 and 2, respectively). Results concerning the isometric phase were similar (372/580; 64.1% of the repetitions). In contrast, in the eccentric phase, the degree of compliance was lower (151/580; 26% of the repetitions in range), with 70.0% of the repetitions performed longer (in time) than expected. However, total TUTs showed a high level of compliance (536/580; 92.4% of the repetitions in range). For the slow abduction scenario (Figure 4), the degree of compliance showed a similar behaviour per phase to the fast scenario, with better compliance in the concentric and isometric phases with respect to the eccentric ones. Even so, the number of repetitions in range for the isometric phase (247/580; 42.6%) and the total phase (410/580; 70.7%) were lower than in the fast scenario. Results concerning the restricted ROM scenario (Figure 5) were in line with the two previous scenarios since the degree of compliance (repetitions in range) was better for concentric (269/580; 46.4%) and isometric (235/580; 40.5%) phases than for the eccentric phase (59/580; 10.2%). Finally, as in the two previous scenarios, the restricted ROM scenario showed better degrees of compliance for concentric (269/580; 46.4%) and isometric (235/580; 40.5%) phases than for the eccentric phase (59/580; 10.2%). These values, alongside the total TUTs (340/580; 58.6%), were, nevertheless, lower than in both previous scenarios. Furthermore, whilst the repetitions out of range tended to be performed in less time than expected for the fast and slow abduction scenarios (179/580; 30.9% and 136/580; 23.4%, respectively), performing beyond the expected time was the general tendency for the restricted ROM scenario (235/580; 40.5%).

#### 3.1.2. Knee Extension

**Figure 6:**
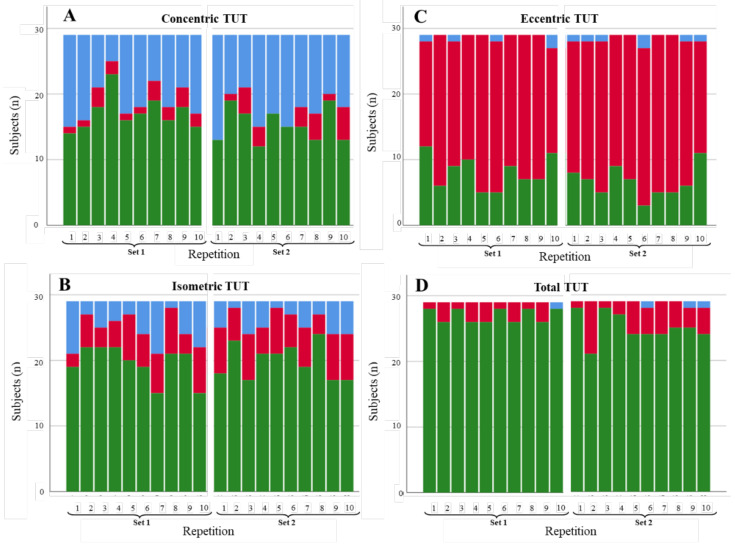
Degree of compliance of TUTs for the fast extension scenario by contraction phase: (**A**) concentric, (**B**) isometric, (**C**) eccentric, and (**D**) total. TUTs: in range = green; performed in less time than expected = blue; performed in more time than expected = red.

**Figure 7:**
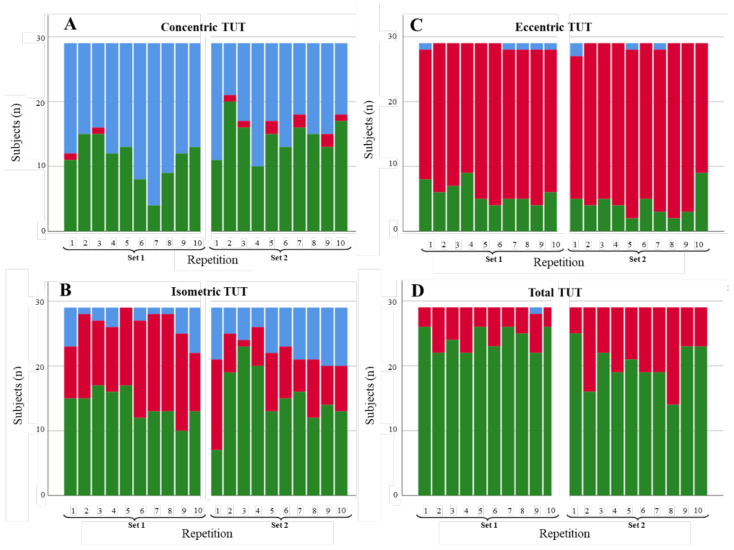
Degree of compliance of TUTs for the slow extension scenario by contraction phase: (**A**) concentric, (**B**) isometric, (**C**) eccentric, and (**D**) total. TUTs: in range = green; performed in less time than expected = blue; performed in more time than expected = red.

**Figure 8:**
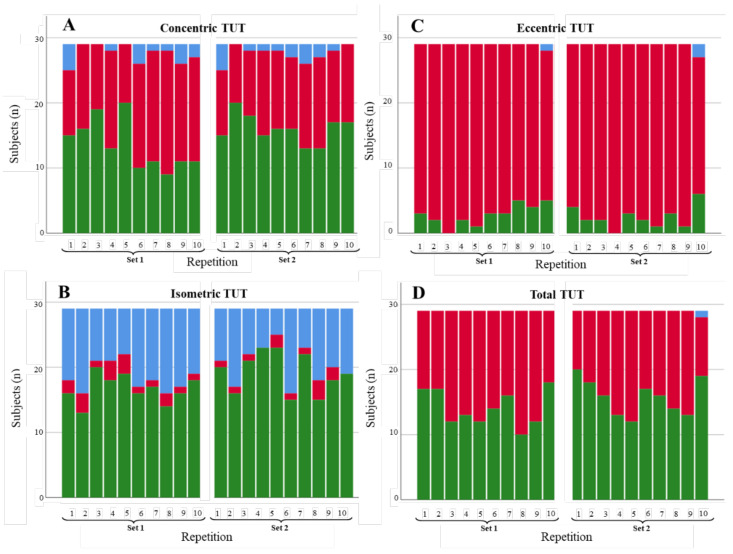
Degree of compliance of the TUTs for the restricted extension ROM scenario by contraction phase: (**A**) concentric, (**B**) isometric, (**C**) eccentric, and (**D**) total. TUTs: in range = green; performed in less time than expected = blue; performed in more time than expected = red.

Figure 6, Figure 7 and Figure 8 illustrate the degree of compliance of TUTs in each repetition per contraction phase for the three knee extension scenarios. Along the same lines as shoulder abduction, the degree of compliance (repetitions in range) in the fast extension scenario was better for concentric (324/580; 55.9%) and isometric (395/580; 68.1%) phases than for the eccentric phase (147/580; 25.3%). Again, the total TUTs showed a high degree of compliance values (520/580; 89.6%). The slow extension scenario showed a similar pattern to the fast scenario across phases (with better degrees of compliance in the concentric and isometric phases than in the eccentric phase), but the values of the degree of compliance were slightly lower, with a number of repetitions with TUTs in the range of 258/580, 44.4%; 293/580, 50.5%; 101/580, 17.4%, respectively. In addition, the isometric phase showed (especially in series 1) a high number of repetitions performed in more time than expected (193/580; 33.3%). Finally, the restricted extension ROM scenario performed slightly differently than the extant two scenarios. Whereas the concentric and isometric phases had, again, better degrees of compliance than the eccentric phase (295/580; 50.9% and 359/580; 61.9% versus 52/580; 8.9%), the percentage of repetitions in range for the total TUTs was lower than in some phases (299/580; 51.5%).

### 3.2. Force

Table 2 shows the mean force values registered in three scenarios for shoulder abduction and knee extension in Newtons (N). For the fast abduction scenario, force at 50% of the concentric movement was 18.03 N, representing 64% of the maximum force (28.14 N), while at 80% of the concentric movement, the force was 23.82 N, representing 85% of the maximum force. The aforementioned results were similar to those obtained in the eccentric contraction phase, although they were slightly lower. Values obtained for the slow scenario were also very close to those obtained for the fast scenario, showing differences below 0.40 N. For the restricted ROM scenario, results were roughly half of the values obtained in the other scenarios. On the other hand, as expected, the force values obtained for the knee scenarios were higher than those obtained in the shoulder abduction scenarios. At 50% and 80% of the concentric movement, the force was, respectively, 70% and 89% of the maximum force (70.12 N). These results were similar to those obtained in the eccentric contraction phase, although they were slightly lower, as in the shoulder abduction scenarios. Values obtained for the slow scenario were also very close to those obtained for the fast scenario, with differences below 1.01 N. Again, the restricted ROM scenario showed force values around half of the values from the other two scenarios.

#### Differences between Estimated and Real Force

Table 3 shows the differences, per exercise and scenario, between the estimated maximum force (i.e., reference values provided by the manufacturer) and the real maximum force obtained. All the obtained maximum force values were lower than the expected values, with differences ranging from 34.1% to 38.5% for the shoulder abduction and from 24.8% to 29.5% for the knee extension. All differences showed large effect sizes (Cohen’s *d* > 2.48).

## 4. Discussion

The aims of this study were to evaluate the degree of compliance with the prescription of TUTs for three different scenarios of two exercises, and the comparison of the force values obtained versus the estimated values. Four important findings emerged: first, the concentric and isometric phases showed a higher degree of compliance for their TUTs than the eccentric phase TUTs for all scenarios of both exercises, whereas the degree of compliance was higher for the total TUT than for the phases’ TUTs; second, the eccentric phase showed a general tendency to be developed for longer time periods than prescribed; third, the fast scenario showed a higher degree of compliance for the isometric phase TUTs and total TUTs than the other two scenarios in both exercises; fourth, the force of the elastic bands tends to be overestimated according to the estimates of the manufacturers.

To our knowledge, this is the first study to examine the degree of compliance on the TUTs of common training exercises with elastic bands per contraction phase. Our findings showed differences in the percentage of repetitions with TUTs in range across the different phases, with a degree of compliance higher for the concentric and isometric phases compared with the eccentric phase for all scenarios and exercises. In addition, the results stemming from the eccentric phase consistently showed how subjects usually tend to perform this phase for a longer time period than prescribed: it may be due to the fact that the eccentric phase corresponds to the return phase and the subjects have to resist the traction force of the elastic band. Thus, the possible fear of performing it faster than prescribed may result in a reactive overaction. Therefore, our results could help health and training professionals who guide exercises with elastic bands, so that they specifically highlight the importance of complying with the TUT for this phase and especially emphasize not performing it slower than the indicated TUT.

On the other hand, the total TUT showed a degree of compliance generally higher than TUTs for each one of the phases. Since previous studies have used this parameter [27,34] instead of TUTs by phases to evaluate compliance, our findings would indicate that the use of the total TUT parameter for this purpose may be an error by masking different degrees of compliance across phases. 

The isometric phase showed a better degree of compliance for the fast scenario than for the slow or restricted scenarios. In all three scenarios, the prescribed TUT for the isometric phase was 2 s, so a difference in the degree of compliance across scenarios is unexpected, given that the TUT remains the same. In addition, except for the slow knee extension, those repetitions in which the TUT was not in range tended to be performed in less time than prescribed, that is, the isometric phase lasted less than 1.8 s. This may be due to the fact that the subjects tend to become fatigued when holding the position and traction of the elastic band, especially in the shoulder, and tend to start the return (i.e., eccentric phase) earlier than prescribed. Our findings would, therefore, suggest paying special attention to maintaining the prescribed TUT of the isometric phase.

On another note, in relation to the force analysis, our findings show a clear overestimation of the force data provided by the manufacturer, since the force obtained was at least 25% less than estimated. Previously, Uchida et al. [25] showed similar differences to ours, under laboratory conditions, in the comparisons of the obtained versus estimated force values, finding difference percentages of 22% for the blue elastic band and 42% for the golden [25]. Thus, our findings confirm that the values proposed by the manufacturer should be used cautiously since the tension (i.e., intensity) applied by the elastic band is actually lower. Furthermore, according to our knowledge, our study is the first to explore and propose values of 50% and 80% of the ROM of two of the most commonly used exercises for training with elastic bands. 

To the best of our knowledge, no previous studies have examined the exercises of shoulder abduction and knee extension with elastic bands in the training scenarios proposed in the literature (i.e., fast or slow execution or performance with restricted ROM). Furthermore, the use of a reference measure to evaluate velocity and tension (linear encoder and force gauge, respectively), and their simultaneous synchronisation could be considered one of the main strengths of the current study. Despite its novel findings, this study was subject to some limitations: first, analyses were conducted on healthy subjects, mainly males, without recent injuries, limiting the generalisation to other populations. Thus, TUTs for fast or slow scenarios could be different for athletes from sports with a predominant use of a particular lower or upper limb (e.g., soccer or baseball, respectively) or between sexes. Additionally, the restricted ROM scenario simulates a situation in which the person has a condition hindering the movement of the segment throughout the ROM. Since we have studied all three scenarios at the same time, we understand that each scenario can be of paramount importance to each target population. Therefore, future studies should examine TUTs in specific populations for which each scenario is the main recommendation. Additionally, measures were made in a controlled environment. Although our study is a first approach to the analysis of the degree of compliance with TUTs and force carried out under the supervision of a physiotherapist, future studies should examine the degree of compliance in a home environment, under no supervision, after familiarisation with the exercises and scenarios.

## 5. Conclusions

Our study provides insight into the degree of compliance with the TUTs for the different phases of two of the most commonly used training exercises with elastic bands, showing that the eccentric phase has a lower degree of compliance than the concentric and isometric phases and that the total TUT would not be advisable to use since it can mask what happened in the different phases. In addition, the analysis of force for the elastic bands used in our study revealed that the values described by the manufacturer are usually overestimated.

## Figures and Tables

**Table 1:** TUT parameters (in seconds) by scenario and phase for shoulder abduction and knee extension.

	Fast Scenario Mean (SD); 95% CI	Slow Scenario Mean (SD); 95% CI	Restricted ROM Scenario Mean (SD); 95% CI
Shoulder ABD			
Concentric time	0.95 (0.12); 0.92–1.02	2.95 (0.14); 2.9–3.01	1.62 (0.17); 1.56–1.69
Isometric time	1.92 (0.22); 1.86–2.03	1.81 (0.12); 1.76–1.85	1.82 (0.19); 1.75–1.89
Eccentric time	1.19 (0.16); 0.95–1.21	3.74 (0.22); 3.65–3.82	2.01 (0.21); 1.93–2.09
Total time	4.07 (0.24); 3.53–4.13	8.5 (0.23); 8.41–8.58	5.45 (0.41); 5.3–5.61
Knee extension			
Concentric time	0.93 (0.11); 0.89–1.05	2.73 (0.18); 2.66–2.79	1.63 (0.15); 1.57–1.68
Isometric time	2.01 (0.21); 1.95–2.08	2.07 (0.17); 2.01–2.13	1.9 (0.17); 1.83–1.96
Eccentric time	1.20 (0.17); 0.96–1.24	3.68 (0.33); 3.56–3.81	1.97 (0.19); 1.9–2.04
Total time	4.15 (0.20); 3.97–4.23	8.48 (0.29); 8.37–8.59	5.49 (0.2); 5.41–5.57

SD = standard deviation; CI = confidence interval; ABD = abduction.

**Table 2:** Force parameters (Newtons) by scenario for shoulder abduction and knee extension.

	Fast Scenario Mean (SD); 95% CI	Slow Scenario Mean (SD); 95% CI	Restricted ROM Scenario Mean (SD); 95% CI
Shoulder abduction			
50% Concentric	18.03 (2.37); 17.13–18.93	17.78 (2.08); 16.98–18.57	13.34 (2.32); 8.72–10.49
80% Concentric	23.82 (2.88); 22.73–24.92	23.43 (2.53); 22.46–24.39	13.34 (3.01); 12.19–14.48
Maximum	28.14 (3.3); 26.89–29.39	27.74 (2.84); 26.66–28.82	15.72 (3.29); 14.47–16.97
80% Eccentric	21.03 (2.56); 20.06–22	21.17 (2.22); 20.32–22.01	11.88 (2.59); 10.9–12.87
50% Eccentric	14.78 (2.02); 14.01–15.55	14.98 (1.78); 14.3–15.66	7.84 (1.81); 7.15–8.52
Knee extension			
50% Concentric	49.36 (2.75); 48.31–50.4	49.64 (3.28); 48.4–50.89	34.67 (3.69); 33.27–36.08
80% Concentric	62.49 (3.37); 61.21–63.77	62.6 (3.97); 61.09–64.11	45.76 (4.49); 44.05–47.46
Maximum	70.12 (4.28); 68.49–71.75	70.96 (5.04); 69.05–72.88	51.33 (4.91); 49.47–53.2
80% Eccentric	55.80 (3.33); 54.53–57.06	56.78 (4.12); 55.21–58.34	41.5 (4.25); 39.88–43.11
50% Eccentric	40.91 (2.51); 39.96–41.87	41.92 (3.03); 40.77–43.07	28.73 (3.26); 27.49–29.97

SD = standard deviation; CI = confidence interval.

**Table 3:** Differences between estimated maximum force (Newtons) and real maximum force (Newtons) per exercise and scenario.

	Estimated Maximum Force Mean (SD); 95% CI	Force Differences (Estimated Force Minus Real Force)	
		Mean (%); 95% CI	Effect Size (95% CI)
Shoulder abduction			
Fast scenario	44.77 (2.89); 43.67/45.87	17.03 (38.1%); 16.35/17.71 *	5.94 (4.66 to 7.70
Slow scenario	45.75 (3.59); 44.39/47.12	17.61 (38.5%); 16.92/18.31 *	5.10 (3.94 to 6.72)
45° scenario	23.85 (3.28); 22.6/25.1	8.13 (34.1%); 7.49/8.78 *	2.48 (1.85 to 2.94)
Knee extension			
Fast scenario	98.43 (5.91); 96.18/100.68	27.47 (27.9%); 25.71/29.22 *	5.01 (4.32 to 5.80)
Slow scenario	99.41 (5.42); 97.35/101.47	29.29 (29.5%); 27.71/30.88 *	6.01 (4.97 to 6.94)
45° scenario	68.22 (5.09); 66.29/70.16	16.89 (24.8%); 15.86/17.92 *	3.38 (2.57 to 4.14)

SD = standard deviation; CI = confidence interval. * Significant differences at *p* < 0.001.

## Data Availability

The datasets generated during and/or analysed during the current study are available from the corresponding authors on reasonable request.
